# Supplementary figures and images for: Unveiling Replication Timing-Dependent Mutational Biases: Mechanistic Insights from Gene Knockouts and Genotoxins Exposures
Source: Int J Mol Sci. 2025 Jul 29;26(15):7307. doi: 10.3390/ijms26157307 (PMC12347494; doi:10.3390/ijms26157307)

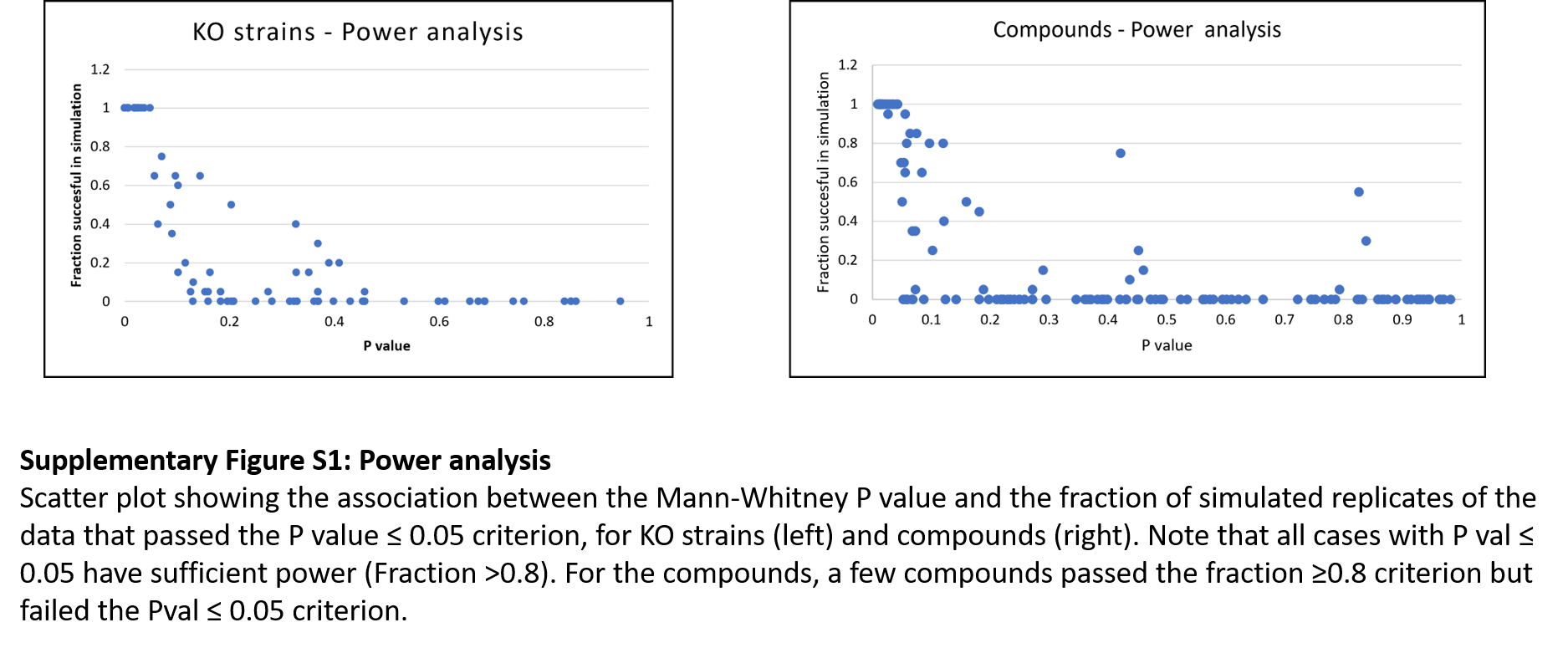

Supplement: Supplementary file 1 [file ijms-26-07307-s001.zip › Supplementary Figure.png]
